# Trends in the incidence and outcome of sepsis using data from a Japanese nationwide medical claims database-the Japan Sepsis Alliance (JaSA) study group-

**DOI:** 10.1186/s13054-021-03762-8

**Published:** 2021-09-16

**Authors:** Taro Imaeda, Taka-aki Nakada, Nozomi Takahashi, Yasuo Yamao, Satoshi Nakagawa, Hiroshi Ogura, Nobuaki Shime, Yutaka Umemura, Asako Matsushima, Kiyohide Fushimi

**Affiliations:** 1 Department of Emergency and Critical Care Medicine, Chiba University Graduate School of Medicine, Chiba, Japan; 2 National Center for Child Health and Development, Critical Care Medicine, Tokyo, Japan; 3 Department of Traumatology and Acute Critical Medicine, Osaka University Graduate School of Medicine, Osaka, Japan; 4 Department of Emergency and Critical Care Medicine, Graduate School of Biomedical and Health Sciences, Hiroshima University, Hiroshima, Japan; 5 Department of Advancing Acute Medicine, Graduate School of Medical Sciences, Nagoya City University, Aichi, Japan; 6 Department of Health Policy and Informatics, Tokyo Medical and Dental University Graduate School of Medical and Dental Sciences, Tokyo, Japan

**Keywords:** Epidemiology, Sepsis-3, Organ dysfunction, ICD-10, Blood culture

## Abstract

**Background:**

Trends in the incidence and outcomes of sepsis using a Japanese nationwide database were investigated.

**Methods:**

This was a retrospective cohort study. Adult patients, who had both presumed serious infections and acute organ dysfunction, between 2010 and 2017 were extracted using a combined method of administrative and electronic health record data from the Japanese nationwide medical claim database, which covered 71.5% of all acute care hospitals in 2017. Presumed serious infection was defined using blood culture test records and antibiotic administration. Acute organ dysfunction was defined using records of diagnosis according to the international statistical classification of diseases and related health problems, 10th revision, and records of organ support. The primary outcomes were the annual incidence of sepsis and death in sepsis per 1000 inpatients. The secondary outcomes were in-hospital mortality rate and length of hospital stay in patients with sepsis.

**Results:**

The analyzed dataset included 50,490,128 adult inpatients admitted between 2010 and 2017. Of these, 2,043,073 (4.0%) patients had sepsis. During the 8-year period, the annual proportion of patients with sepsis across inpatients significantly increased (slope = + 0.30%/year, *P* < 0.0001), accounting for 4.9% of the total inpatients in 2017. The annual death rate of sepsis per 1000 inpatients significantly increased (slope = + 1.8/1000 inpatients year, *P* = 0.0001), accounting for 7.8 deaths per 1000 inpatients in 2017. The in-hospital mortality rate and median (interquartile range) length of hospital stay significantly decreased (*P* < 0.001) over the study period and were 18.3% and 27 (15–50) days in 2017, respectively.

**Conclusions:**

The Japanese nationwide data indicate that the annual incidence of sepsis and death in inpatients with sepsis significantly increased; however, the annual mortality rates and length of hospital stay in patients with sepsis significantly decreased. The increasing incidence of sepsis and death in sepsis appear to be a significant and ongoing issue.

**Supplementary Information:**

The online version contains supplementary material available at 10.1186/s13054-021-03762-8.

## Background

It is estimated that, annually, approximately 48.9 million people worldwide develop sepsis, and of these, 11 million (22.5%) die [1]. Multicenter studies have revealed the characteristics and outcomes of Sepsis-3 [2–4]; however, limited epidemiological investigational studies using a nationwide database have been performed [5]. Japan, which has universal public health care, developed a comprehensive reimbursement system of medical cost, named the diagnosis procedure combination (DPC) system, in 2003 [6]. The claims-based database has data on diagnosis, examination, and treatment; data from this database have been used in epidemiological studies of brain, orthopedic, and abdominal diseases and cancer [7–10], but no studies have used these data with regard to sepsis.

Between 1979 and 2012 in North America and Europe, epidemiological sepsis studies documented that mortality decreased; however, the proportion of patients with sepsis across inpatients and the number of deaths in sepsis increased [11–14]. In contrast, a recent Global Burden of Diseases study in the period of 1990–2017 reported a decreased proportion of patients with sepsis across inpatients and decreased number of deaths in sepsis [1], which is inconsistent with previous studies. Recent trends in sepsis may be changing.

Thus, in the current study, the diagnosis of sepsis was defined as a patient who underwent blood culture with a new antibiotic treatment and presented acute organ dysfunction, which is linked with the third International Consensus Definitions for Sepsis and Septic Shock (Sepsis-3) [15]. We tested the hypothesis that there is a trend for an increased incidence of sepsis and death in sepsis in the aging society of Japan using a large nationwide claims-based database including data on over 50 million inpatients in 2010–2017. The primary outcomes were annual incidence of sepsis and death in sepsis per 1000 inpatients.

## Materials and methods

### Study cohorts

This retrospective cohort study used Japanese nationwide data from the medical reimbursement system for acute care, DPC. Our DPC data, which were obtained from 1237 acute care hospitals, is the largest nationwide acute care dataset in Japan; it covered 71.5% of the total acute care hospitals in 2017 [6]. We extracted all adult patients, who aged ≥ 20 years as previously reported [3], admitted to hospitals between 2010 and 2017 in the DPC data. The exclusion criterion of the study was this age limitation (< 20 years) only. From the adult inpatient cohort, we extracted patients who had both presumed serious infections and acute organ dysfunction. We described definition of presumed serious infections and acute organ dysfunction in the following Definitions section. The Institutional Review Board of Chiba University Graduate School of Medicine has approved the study (approval number, 3429). Because data were anonymized, the requirement to obtain any informed consent from individual patients was waived.

### Data collection and definitions

Data available in the database included patient’s age, sex, admission and discharge dates, discharge status (death/survival), intensive care unit (ICU) admission (including emergency room and coronary care unit); primary diagnosis on admission, comorbidities on admission, and post-hospitalization complications coded using the international statistical classification of diseases and related health problems, 10th revision (ICD-10) (Additional file 1: Table S1) [16]; procedures including mechanical ventilation, oxygen therapy, and renal replacement therapy; therapeutic drugs used during hospitalization including vasoactive agents including dopamine, dobutamine, norepinephrine, or epinephrine, and antimicrobial, on a daily basis; examinations such as blood culture collection. Blood culture results (positive or negative) and laboratory data to calculate SOFA score were not included. The focus of infection was extracted by referring to ICD-10 codes for presumed foci of infection (Additional file 1: Table S2) [17]. All clinical data were recorded by the attending physicians at the time of hospital discharge.

### Sepsis

**Fig. 1:**
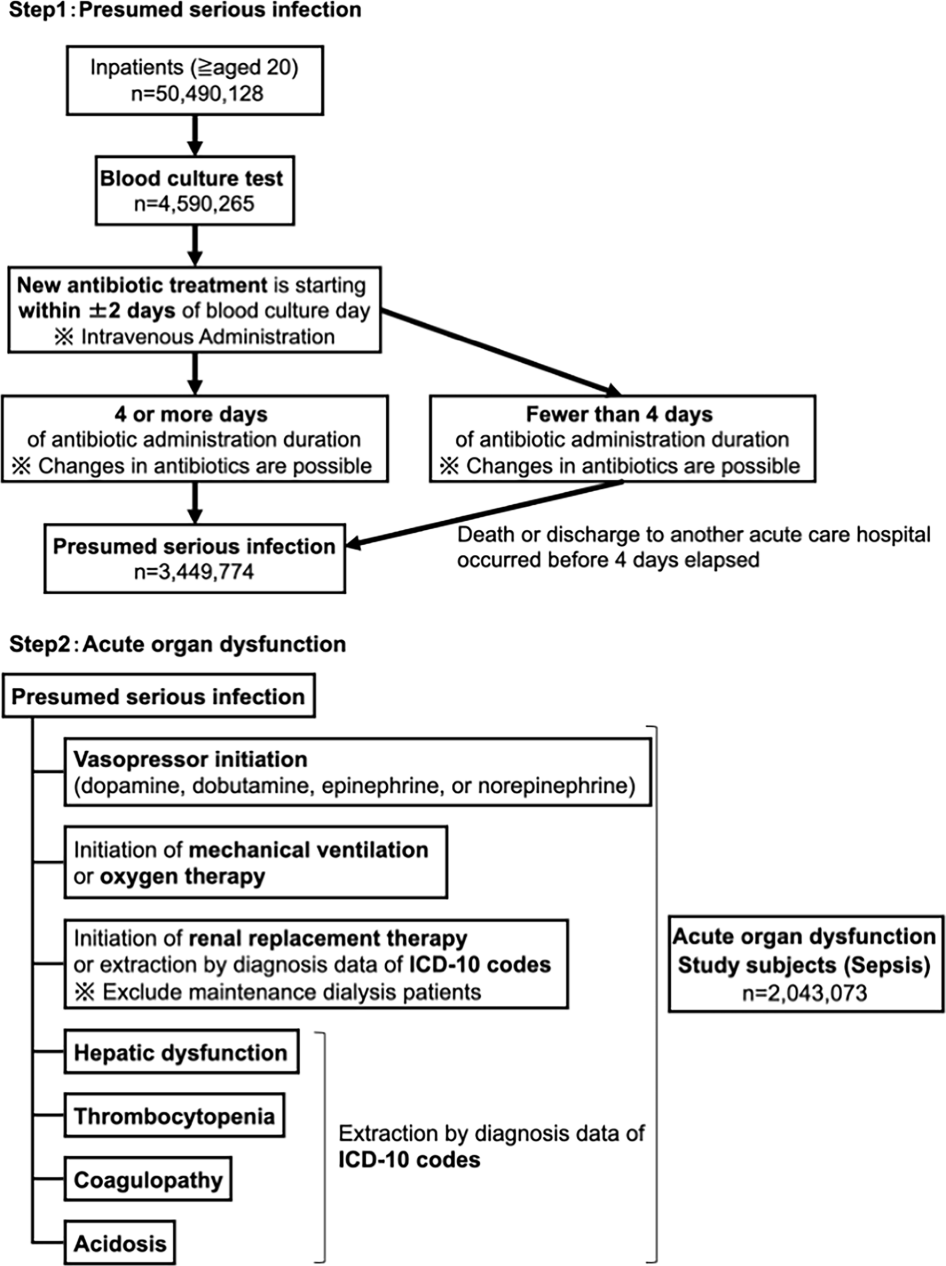
Flow diagram of the study population. In total, 50,490,128 inpatients were enrolled in the study between 2010 and 2017. Of those, 4,590,265 patients underwent a blood culture test. Of these, 3,449,774 patients received antibiotic treatment for ≥ 4 days within ± 2 calendar days of the blood culture draw. Of these, 2,043,073 patients had acute organ dysfunction; these patients were analyzed as study subjects (sepsis)

Based on a previous epidemiological study in the United States (US) after the publication of Sepsis-3 [3], we extracted septic patients who had both presumed serious infections and acute organ dysfunction using a combined method of administrative and electronic health record (EHR) data from the Japanese nationwide medical claim database (Fig. 1).

As previously reported [3], presumed serious infections were defined through blood culture collection records and administration of new antibiotics for four or more days. The first day of antibiotic administration was required to occur within ± 2 calendar days of blood culture collection. Four or more antibiotic days, including at least one intravenous antibiotic, were required. Fewer than 4 days of antibiotic administration duration were allowed if death occurred before the 4 days elapsed.

Laboratory data of creatinine, bilirubin, and platelet counts were not included in the DPC data. Therefore, we extracted patients with acute organ dysfunction using diagnosis codes and organ-specific treatment data. Information regarding acute organ dysfunction was extracted using diagnostic data from the ICD-10 codes for hepatic dysfunction, thrombocytopenia/coagulopathy, and acidosis (Additional file 1: Table S3) [18] as well as data regarding organ support including initiation of vasopressors, mechanical ventilation/oxygen therapy, or renal replacement therapy for circulatory dysfunction, respiratory dysfunction, and renal dysfunction. We excluded maintenance dialysis in patients with end-stage renal disease from renal replacement therapy.

Community-onset sepsis was defined as sepsis within 48 h of hospitalization [19], i.e., cultures/antibiotics initiated with 48 h of presentation to hospital.

### Statistical analysis

The primary outcomes were the annual proportion of patients with sepsis across inpatients and deaths in sepsis per 1000 inpatients overall (hereafter referred to as deaths in sepsis per 1000 inpatients) [12]. The secondary outcomes were in-hospital mortality rate and length of hospital stay in patients with sepsis. Regarding to the definition of in-hospital death from sepsis, we did not use specific cut-offs like 28 days or 90 days. Individual persons were used as the unit of analysis for in-hospital mortality and deaths in sepsis per 1000 inpatients. Patients with repeat hospitalizations were excluded from the analysis of in-hospital mortality and deaths in sepsis per 1000 inpatients. The hospital admission was used as the unit of analysis for length of hospital stay in patients with sepsis and proportion of patients with sepsis across inpatients. The annual changes were analyzed using linear regression or the Cochran–Armitage test for trends in categorical data. The methods of trend analysis applied to the 8 individual calendar years. Subgroup analyses were conducted in three groups using standard criteria for the elderly as previously reports [18, 20, 21]. Additionally, subgroups by dividing into units of 10 years were analyzed. The current study used the medical claim database, which included no data as missing values. Thus, no imputation was performed.

Variables such as length of hospital and ICU stay were expressed as both mean/ standard deviation (SD) and median/interquartile range (IQR) to compare with those in previous reports. Non-normally distributed variables such as age and the hospital day of the blood culture draw were expressed as median/IQR. *P *values < 0.05 were considered statistically significant. SQL (mariadb v10.4.17) and pandas (v1.0.5) in Python (v3.9.0) and Prism software (GraphPad Prism 8; GraphPad Software Inc., San Diego, CA, US) were used for data manipulation and statistical analysis.

## Results

The analyzed dataset included 50,490,128 adult inpatients in total during the 8-year period between 2010 and 2017. Of these, 2,043,073 (4.0%) patients had sepsis, 70.2% of which were community-onset (Table 1; annual data, Additional file 1: Table S4). The age of patients with sepsis significantly increased (*P* = 0.0005; annual data, Additional file 1: Table S4). Major comorbidities included malignant tumors (34.9%), hypertension (26.3%), and diabetes mellitus (21.8%), with the number of patients with hypertension or diabetes mellitus increasing significantly every year (*P* < 0.01; annual data, Additional file 1: Table S4). Respiratory infections were the most common source of infection (41.0%), and significantly increased (*P* < 0.0001; annual data, Additional file 1: Table S4 and S5). Regarding acute organ dysfunction, patients with respiratory failure who used mechanical ventilation/oxygen therapy were the most common, accounting for 82.3% (293,026 patients) in 2017 (Additional file 1: Table S6). 17.1% of patients with sepsis required ICU admission. The median (IQR) length of hospital stay was 29 (16–55) days and the in-hospital mortality rate was 20.1%.

**Table 1:** Demographics and clinical characteristics of patients with sepsis

Screened total inpatients, *n*	50,490,128
Extracted sepsis, *n*	2,043,073
Community-onset sepsis, *n* (%)	1,433,603 (70.2)
Age, year ^a^	76 (66–84)
Female, *n* (%)	884,831 (41.4)
Comorbidity	
Malignant tumor, *n* (%)	712,219 (34.9)
Hypertension, *n* (%)	537,818 (26.3)
Diabetes mellitus, *n* (%)	445,438 (21.8)
Heart failure, *n* (%)	376,676 (18.4)
Cerebrovascular disease, *n* (%)	292,637 (14.3)
Ischemic heart disease, *n* (%)	215,868 (10.6)
Chronic respiratory disease, *n* (%)	179,898 (8.8)
Chronic renal failure, *n* (%)	79,362 (3.9)
Focus of infection (*n* = 1,275,988)	
Respiratory, *n* (%)	523,057 (41.0)
Urogenital, *n* (%)	194,799 (15.3)
Abdominal, *n* (%)	147,409 (11.6)
Bone and soft tissue, *n* (%)	70,577 (5.5)
Meninges/brain/spinal cord, *n* (%)	25,984 (2.0)
Heart, *n* (%)	12,139 (1.0)
Sexually transmitted disease, *n* (%)	3719 (0.3)
Blood, *n* (%)	3560 (0.3)
Unknown, *n* (%)	294,745 (23.0)
The hospital day of the blood culture draw, day ^a^	1 (1–2)
Length of antibiotic treatment, days	
Mean (SD)	18.7 (21.4)
Median (IQR)	12 (8–21)
Length of hospital stay, days	
Mean (SD)	45.7 (104.9)
Median (IQR)	29 (16–55)
ICU admission, *n* (%)	350,365 (17.1)
Length of ICU stay, days	
Mean (SD)	6.9 (6.2)
Median (IQR)	5 (2–11)
In-hospital mortality, *n* (%) ^b^	363,363 (20.1)

*ICU* intensive care unit, *SD* standard deviation, *IQR* interquartile range

^a^The data shown represent the median value along with the interquartile range

^b^After excluding repeat hospitalizations, total number of patients with sepsis was 1,808,113

**Fig. 2:**
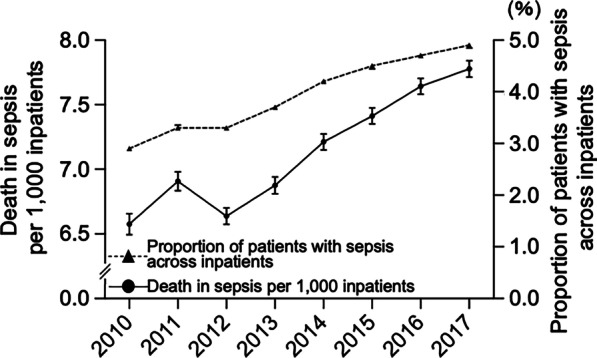
Annual change in deaths per 1000 inpatients and proportion of patients with sepsis across inpatients. Deaths per 1000 inpatients: + 1.8/y [95% CI + 1.2 to + 2.3], *R*^2^ = 0.90, *P* = 0.0003. Proportion of patients with sepsis across inpatients: + 0.30%/y [95% CI + 0.25% to + 0.34%], *R*^2^ = 0.98, *P* < 0.0001. Error bars indicate 95% CI. *CI* confidence interval

The annual proportion of patients with sepsis across inpatients significantly increased (*R*^2^ = 0.98, slope = + 0.30%/year, *P* < 0.0001; Fig. 2). The annual number of patients with sepsis was 355,833 (4.9% of total inpatients) in 2017 (Additional file 2: Fig. S1). The annual deaths in sepsis per 1000 inpatients significantly increased (*R*^2^ = 0.90, slope = + 1.8/1000 inpatients year, *P* = 0.0003; Fig. 2), accounting for 7.8 annual deaths in sepsis per 1000 inpatients in 2017. The annual number of deaths in patients with sepsis was 56,905 in 2017 (a 2.3-fold increase compared to 2010) (Additional file 2: Fig. S1).

**Fig. 3:**
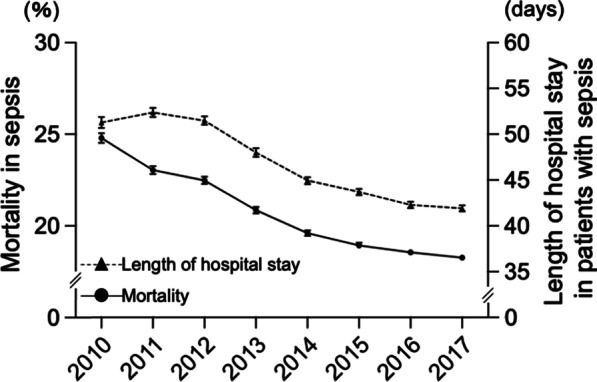
Annual change in the in-hospital mortality rate and mean length of hospital stay. In-hospital mortality: − 0.95%/y [95% CI − 1.17% to − 0.73%], *R*^2^ = 0.95, *P* < 0.0001. Length of hospital stay: − 1.70 days/y [95% CI − 2.21 to − 1.19], *R*^2^ = 0.92, *P* = 0.0002. Error bars indicate 95% CI. *CI* confidence interval

**Fig. 4:**
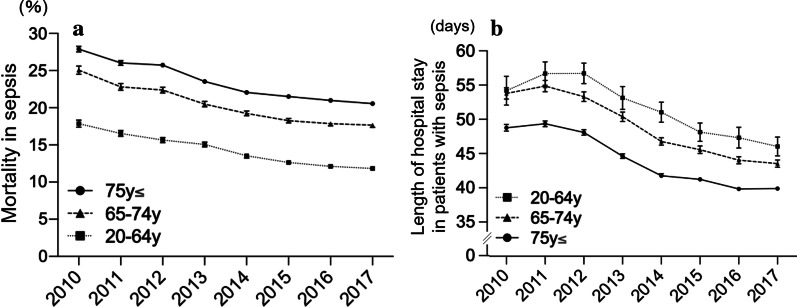
**a** Annual change in the in-hospital mortality rate according to the age subgroups. ≥ 75 years: − 1.08%/y [95% CI − 1.33% to − 0.82%], *R*^2^ = 0.95, *P* < 0.0001. 65–74 years: − 1.08%/y [95%CI − 1.34% to − 0.81%], *R*^2^ = 0.94, *P* < 0.0001. 20–64 years: − 0.89%/y [95%CI − 1.04% to − 0.75%], *R*^2^ = 0.98, *P* < 0.0001. Error bars indicate 95% CI. *CI* confidence interval. **b** Annual change in the length of hospital stay according to the age subgroups. ≥ 75 years: − 1.58%/y [95% CI − 2.06% to − 1.11%], R^2^ = 0.92, *P* = 0.0002. 65–74 years: − 1.82%/y [95% CI − 2.31% to − 1.33%], *R*^2^ = 0.93, *P* = 0.0001. 20–64 years: − 1.57%/y [95% CI − 2.24% to − 0.89%], *R*^2^ = 0.84, *P* = 0.0013. Error bars indicate 95% CI. *CI* confidence interval

The in-hospital mortality rate and length of hospital stay in sepsis significantly decreased (*R*^2^ = 0.95, slope = − 0.95%/year, *P* < 0.0001 and *R*^2^ = 0.92, slope = − 1.70 days/year, *P* = 0.0002, respectively; Fig. 3). In 2017, the in-hospital mortality rate was 18.3%, and the mean length of hospital stay was 41.9 days in 2017. Repeating analysis in the age subgroups revealed that the decreased in the in-hospital mortality rate and hospital stay were significant in all age subgroups (*P* < 0.0001 and *P* < 0.01, respectively; Fig. 4a and 4b) (Additional file 3: Fig. S2a and Additional file 4: Fig. S2b). Additional analysis by dividing into units of 10 years also revealed the decreasing trend of in-hospital mortality in all the subcategories; old groups have more downslope compared to young groups (Additional file 3: Fig. S2a).

## Discussion

In the present nationwide sepsis study, the annual incidence of sepsis and death in inpatients with sepsis significantly increased during the 8-year study period (2010–2017) in Japan. The in-hospital mortality rate and length of hospital stay significantly decreased during this period.

The proportion of patients with sepsis across inpatients increased from 0.99 to 2.38% during 2000–2007 in the US [12], from 1.2 to 2.7% during 2005–2014 in the US [22], from 1.21 to 1.54% during 2007–2013 in Germany [23], and from 1.3 to 2.1% during 2008–2012 in Spain [24]. In accordance with these results, we found the proportion of patients with sepsis across inpatients significantly increased from 2.9 to 4.9% between 2010 and 2017. The incidence of sepsis in the present study is relatively higher compared to those in the US/Europe. Differences in study region or methods of sepsis selection potentially provides the differences. In line with these frequencies per inpatients, population-level incidence rates for sepsis are also increasing but different among regions. In the US, those increased from 346/100,000 to 436/100,000 (2008–2012) [5], in South Korea from 173.8/100,000 to 233.6/100,000 (2007–2016) [25], and from 637.8/100,000 to 772.1/100,000 in Taiwan (2002–2012) [26].

Previous studies of severe sepsis using the definition prior to the publication of “Sepsis-3” reported an increased number of deaths in sepsis. In 2011, Kumar et al. reported an increase in the number of deaths in inpatients with sepsis between 2000 and 2007 (8 years) in the US, from 4.0 deaths/1000 inpatients in 2000 to 6.5 deaths/1000 inpatients in 2007; the annual absolute number of deaths increased 1.8 times (213,124 deaths in 2007) [12]. In 2012, Lagu et al. reported an increased number of deaths between 2003 and 2007 (5 years) in the US, from 4.9 deaths/1000 inpatients in 2003 to 6.3 deaths/1000 inpatients in 2007; the annual absolute number of deaths increased 1.3 times (207,427 deaths in 2007) [12, 27]. In accordance with these results, we found a significant increase in the number of annual deaths in inpatients with sepsis (Slope =  + 1.8/1000 inpatients year, *P* = 0.0003), an absolute increase of 2.3 times (56,905 deaths in 2017). In contrast, a recent Global Burden of Disease study, which extracted sepsis using the ICD codes, showed a decreased number of deaths in sepsis worldwide [1]. Different regions (worldwide vs. Japan) or different extraction methods (diagnosis data only vs. diagnosis plus examination plus treatment data) may yield different results.

Substantial studies reported that the annual mortality of sepsis decreased. A meta-analysis of 36 multicenter trials of severe sepsis showed a decrease in mortality from 46.9% between 1991 and 1995 to 29% between 2006 and 2009 [28]. A US nationwide study showed decreased mortality of severe sepsis from 39 to 27% during 2000–2007 [12]. Similar trends have been observed in Asian countries, with mortality rate decreasing from 30.9% in 2007 to 22.6% in 2016 in South Korea [25] and from 27.8% in 2002 to 22.8% in 2012 in Taiwan [26]. In accordance with these findings, we found that rate of mortality in sepsis decreased from 25.0 to 18.3% between 2010 and 2017. Furthermore, in age subgroup analysis by dividing into units of 10 years, the elderly had more annual improvement of mortality compared to the young (≥ 80 years: − 1.11%/years, *P* < 0.0001; 20–29 years: − 0.31%/years, *P* = 0.011). In Rhee’s study in the US [3], 54.7% of patients with sepsis required ICU, while only 17% of those required ICU in the present study. Japan has a low number of ICU bed (5 per 100,000 people) compared to the US (20 per 100,000 people) [29], which may reflect this low frequency of ICU admission. The length of hospital stay also decreased during the study period. In an epidemiological study of sepsis in Spain, the average length of hospital stay decreased from 15.7 to 14.0 days during 2000–2013 [30]. In the US studies, the average length of hospital stay decreased from 14.0 to 12.5 days during 2000–2007 [12] and 16.1 to 10.7 days during 2005–2014 [22]. In accordance with these findings, we found that the average length of hospital stay decreased from 51.3 to 41.9 days between 2010 and 2017. These improvements in mortality and length of hospital stay in sepsis may be attributed to the widespread use of the international and Japanese sepsis guidelines, which potentially contribute to improvements in the recognition of sepsis, faster treatment, and improvement in care for critically ill patients [31–34]. In comparison of length of hospital stay in patients with acute care among 34 American, European, and Asian countries in 2019, Japan had extremely long hospital stays (average 16.0 days) compared to all other countries (average ranged from 4.1 to 9.3 days) [35]. In Rhee’s study of patients with sepsis in the US, the median (IQR) hospital length of stay was 10 days (8–12) in 2014 [3]. A Japanese multicenter observational study of severe sepsis (2016–2017, *n* = 1184) reported long hospital stays [median (IQR) 24 (12–46)] [4]. In accordance with these, we found the length of hospital stay in patients with sepsis was long [median (IQR) 29 (16–55)]. Japanese health care system may involve these longer hospital stays [36].

We acknowledge that our study has some limitations. First, the DPC data did not include laboratory data. Since the definition of sepsis requires laboratory data, including blood levels of creatinine and bilirubin and platelet counts, our extraction approach using diagnosis coding and treatment data on organ supports may underestimate the incidence compared to a more stringent extraction approach using laboratory data according to Sepsis-3. On the other hand, a recent report [37] found that the Rhee criteria [3] using a combination of culture and antibiotic orders approach is significantly less sensitive but more specific compared approaches to defining suspected infection via EHR data. We would expect the current study using suspected infection definition to be relatively specific and identify a more severely ill population. Second, due to the unavailability of blood lactate data, we could not analyze septic shock according to Sepsis-3. Third, the current study is retrospective using administrative database. Fourth, although the DPC is the largest nationwide dataset, the datasets only covered 71.5% of all acute care hospitals. Fifth, the median age of 76 years with the high prevalence of malignancy (35%) limits the generalizability of this study. Sixth, the median length of antibiotic treatment analyzed data between 2010 and 2017 in the present study was 12 days, which was relatively longer than those (median 10 days) in a recent randomized control trial of sepsis in between 2017 and 2019 [38]. This study included substantial patients with respiratory infections (41.0%) and elderly (≥ 65 years old, 78.6%). Since respiratory infections in the elderly have repeated fevers and longer antibiotic treatment [39, 40], these characteristics may partially contribute to the long duration of antibiotic treatment. Older study period may influence the difference in the longer durations of antibiotic treatment. In addition, the incidence of the blood stream infection was low (< 1%). Since we could not obtain blood culture results, we defined the blood stream infection in the diagnosis coding approach, which may underestimate the incidence of the blood stream infection. Seventh, the value presented in the age subgroup analysis related to in-hospital mortality rate was actually a crude rate without any adjustment for patient severity or other factors due to data availability.

## Conclusions

The in-hospital mortality rate and length of hospital stay in patients with sepsis significantly improved between 2010 and 2017; however, the incidence of sepsis and death in inpatients with sepsis has been increasing annually in Japan. Sepsis appears to be a significant and ongoing issue. Health systems may consider countermeasure for reduce sepsis incidence.

## Supplementary Information

**Additional file 1: Table S1.** Comorbidity categories with corresponding ICD-10. **Table S2.**. Focus of infection with corresponding ICD-10. **Table S3.** Acute organ dysfunction categories with corresponding ICD-10. **Table S4.** Demographics and clinical characteristics of patients with sepsis. **Table S5.** Focus of infection. **Table S6.** Organ support or dysfuntion.

**Additional file 1: Figure S1.** Annual change in the number of patients with sepsis and deaths in sepsis.

**Additional file 1: Figure S2a.** Annual change in the in-hospital mortality rate according to the age subgroups by dividing into units of 10 years.

**Additional file 1: Figure S2b.** Annual change in the length of hospital stay according to the age subgroups by dividing into units of 10 years.

## Data Availability

The datasets analyzed during the current study are available with the corresponding author on reasonable request.

## Abbreviations

DPC: Diagnosis procedure combination
EHR: Electronic health record
ICD-10: International statistical classification of diseases and related health problems 10th revision
IQR: Interquartile range
SD: Standard deviation
Sepsis-3: Third international consensus definitions for sepsis and septic shock
US: United States

## References

[1] Rudd KE, Johnson SC, Agesa KM, Shackelford KA, Tsoi D, Kievlan DR, Colombara DV, Ikuta KS, Kissoon N, Finfer S (2020). Global, regional, and national sepsis incidence and mortality, 1990–2017: analysis for the global burden of disease study. Lancet.

[2] Shankar-Hari M, Harrison DA, Rubenfeld GD, Rowan K (2017). Epidemiology of sepsis and septic shock in critical care units: comparison between sepsis-2 and sepsis-3 populations using a national critical care database. Br J Anaesth.

[3] Rhee C, Dantes R, Epstein L, Murphy DJ, Seymour CW, Iwashyna TJ, Kadri SS, Angus DC, Danner RL, Fiore AE (2017). Incidence and trends of sepsis in US hospitals using clinical vs claims data, 2009–2014. JAMA.

[4] Abe T, Ogura H, Shiraishi A, Kushimoto S, Saitoh D, Fujishima S, Mayumi T, Shiino Y, Nakada TA, Tarui T (2018). Characteristics, management, and in-hospital mortality among patients with severe sepsis in intensive care units in Japan: the FORECAST study. Crit Care (Lond Engl).

[5] Fleischmann C, Scherag A, Adhikari NK, Hartog CS, Tsaganos T, Schlattmann P, Angus DC, Reinhart K (2016). Assessment of global incidence and mortality of hospital-treated sepsis. Current estimates and limitations. Am J Respir Crit Care Med.

[6] Yamana H, Moriwaki M, Horiguchi H, Kodan M, Fushimi K, Yasunaga H (2017). Validity of diagnoses, procedures, and laboratory data in Japanese administrative data. J Epidemiol.

[7] Moroi R, Tarasawa K, Shiga H, Yano K, Shimoyama Y, Kuroha M, Kakuta Y, Fushimi K, Fujimori K, Kinouchi Y (2020). Efficacy of urgent colonoscopy for colonic diverticular bleeding: a propensity score-matched analysis using a nationwide database in Japan. J Gastroenterol Hepatol.

[8] Mine Y, Fujino Y, Sabanai K, Muramatsu K, Otani M, Kubo T, Fushimi K, Matsuda S (2020). Effectiveness of regional clinical pathways on postoperative length of stay for hip fracture patients: a retrospective observational study using the Japanese diagnosis procedure combination database. J Orthop Sci.

[9] Kobori S, Kubo T, Otani M, Muramatsu K, Fujino Y, Adachi H, Horiguchi H, Fushimi K, Matsuda S (2017). Coexisting infectious diseases on admission as a risk factor for mechanical ventilation in patients with Guillain-Barré syndrome. J Epidemiol.

[10] Shinjo D, Matsumoto K, Terashima K, Takimoto T, Ohnuma T, Noguchi T, Fushimi K (2019). Volume effect in paediatric brain tumour resection surgery: analysis of data from the Japanese national inpatient database. Eur J Cancer.

[11] Martin GS, Mannino DM, Eaton S, Moss M (2003). The epidemiology of sepsis in the United States from 1979 through 2000. N Engl J icine.

[12] Kumar G, Kumar N, Taneja A, Kaleekal T, Tarima S, McGinley E, Jimenez E, Mohan A, Khan RA, Whittle J (2011). Nationwide trends of severe sepsis in the 21st century (2000–2007). Chest.

[13] Kaukonen KM, Bailey M, Suzuki S, Pilcher D, Bellomo R (2014). Mortality related to severe sepsis and septic shock among critically ill patients in Australia and New Zealand, 2000–2012. JAMA.

[14] Rhee C, Gohil S, Klompas M (2014). Regulatory mandates for sepsis care–reasons for caution. N Engl J Med.

[15] Singer M, Deutschman CS, Seymour CW, Shankar-Hari M, Annane D, Bauer M, Bellomo R, Bernard GR, Chiche JD, Coopersmith CM (2016). The third international consensus definitions for sepsis and septic shock (sepsis-3). JAMA.

[16] https://www.who.int/standards/classifications/classification-of-diseases. Accessed 31 Aug 2021.

[17] Jeganathan N, Yau S, Ahuja N, Otu D, Stein B, Fogg L, Balk R (2017). The characteristics and impact of source of infection on sepsis-related ICU outcomes. J Crit Care.

[18] Rhee C, Murphy MV, Li L, Platt R, Klompas M (2015). Improving documentation and coding for acute organ dysfunction biases estimates of changing sepsis severity and burden: a retrospective study. Crit Care (Lond Engl).

[19] https://www.cdc.gov/sepsis/clinicaltools/index.html. Accessed 31 Aug 2021.

[20] Chou HL, Han ST, Yeh CF, Tzeng IS, Hsieh TH, Wu CC, Kuan JT, Chen KF (2016). Systemic inflammatory response syndrome is more associated with bacteremia in elderly patients with suspected sepsis in emergency departments. Medicine (Baltimore).

[21] Guidet B, Leblanc G, Simon T, Woimant M, Quenot JP, Ganansia O, Maignan M, Yordanov Y, Delerme S, Doumenc B (2017). Effect of systematic intensive care unit triage on long-term mortality among critically ill elderly patients in france: a randomized clinical trial. JAMA.

[22] Rubens M, Saxena A, Ramamoorthy V, Das S, Khera R, Hong J, Armaignac D, Veledar E, Nasir K, Gidel L (2020). Increasing Sepsis Rates in the United States: Results From National Inpatient Sample, 2005 to 2014. J Intensive Care Med.

[23] Fleischmann C, Thomas-Rueddel DO, Hartmann M, Hartog CS, Welte T, Heublein S, Dennler U, Reinhart K (2016). Hospital incidence and mortality rates of sepsis. Dtsch Arztebl Int.

[24] Yébenes JC, Ruiz-Rodriguez JC, Ferrer R, Clèries M, Bosch A, Lorencio C, Rodriguez A, Nuvials X, Martin-Loeches I, Artigas A (2017). Epidemiology of sepsis in Catalonia: analysis of incidence and outcomes in a European setting. Ann Intensive Care.

[25] Oh SY, Cho S, Kim GH, Jang EJ, Choi S, Lee H, Ryu HG (2019). Incidence and outcomes of sepsis in Korea: a nationwide cohort study from 2007 to 2016. Crit Care Med.

[26] Lee CC, Yo CH, Lee MG, Tsai KC, Lee SH, Chen YS, Lee WC, Hsu TC, Lee SH, Chang SS (2017). Adult sepsis—a nationwide study of trends and outcomes in a population of 23 million people. J Infect.

[27] Lagu T, Rothberg MB, Shieh MS, Pekow PS, Steingrub JS, Lindenauer PK (2012). Hospitalizations, costs, and outcomes of severe sepsis in the United States 2003 to 2007. Crit Care Med.

[28] Stevenson EK, Rubenstein AR, Radin GT, Wiener RS, Walkey AJ (2014). Two decades of mortality trends among patients with severe sepsis: a comparative meta-analysis*. Crit Care Med.

[29] Ohbe H, Sasabuchi Y, Kumazawa R, Matsui H, Yasunaga H (2021). Intensive care unit occupancy in Japan, 2015–2018: a nationwide inpatient database study. J Epidemiol.

[30] Alvaro-Meca A, Jimenez-Sousa MA, Micheloud D, Sanchez-Lopez A, Heredia-Rodriguez M, Tamayo E, Resino S (2018). Epidemiological trends of sepsis in the twenty-first century (2000–2013): an analysis of incidence, mortality, and associated costs in Spain. Popul Health Metr.

[31] Rhodes A, Evans LE, Alhazzani W, Levy MM, Antonelli M, Ferrer R, Kumar A, Sevransky JE, Sprung CL, Nunnally ME (2017). Surviving sepsis Campaign: international guidelines for management of sepsis and septic shock: 2016. Intensive Care Med.

[32] Levy MM, Dellinger RP, Townsend SR, Linde-Zwirble WT, Marshall JC, Bion J, Schorr C, Artigas A, Ramsay G, Beale R (2010). The surviving sepsis campaign: results of an international guideline-based performance improvement program targeting severe sepsis. Crit Care Med.

[33] Cannon CM, Holthaus CV, Zubrow MT, Posa P, Gunaga S, Kella V, Elkin R, Davis S, Turman B, Weingarten J (2013). The GENESIS project (GENeralized Early Sepsis Intervention Strategies): a multicenter quality improvement collaborative. J Intensive Care Med.

[34] Miller RR, Dong L, Nelson NC, Brown SM, Kuttler KG, Probst DR, Allen TL, Clemmer TP (2013). Multicenter implementation of a severe sepsis and septic shock treatment bundle. Am J Respir Crit Care Med.

[35] https://data.oecd.org/healthcare/length-of-hospital-stay.htm. Accessed 31 Aug 2021.

[36] https://www.oecd.org/publications/oecd-reviews-of-health-care-quality-japan-2015-9789264225817-en.htm. Accessed 31 Aug 2021.

[37] Churpek MM, Dumanian J, Dussault N, Bhavani SV, Carey KA, Gilbert ER, Arain E, Ye C, Winslow CJ, Shah NS (2021). Determining the electronic signature of infection in electronic health record data. Crit Care Med.

[38] Kyriazopoulou E, Liaskou-Antoniou L, Adamis G, Panagaki A, Melachroinopoulos N, Drakou E, Marousis K, Chrysos G, Spyrou A, Alexiou N (2021). Procalcitonin to reduce long-term infection-associated adverse events in sepsis. A randomized trial. Am J Respir Crit Care Med.

[39] Ito I, Kadowaki S, Tanabe N, Haruna A, Kase M, Yasutomo Y, Tsukino M, Nakai A, Matsumoto H, Niimi A (2010). Tazobactam/piperacillin for moderate-to-severe pneumonia in patients with risk for aspiration: comparison with imipenem/cilastatin. Pulm Pharmacol Ther.

[40] Teramoto S, Fukuchi Y, Sasaki H, Sato K, Sekizawa K, Matsuse T (2008). High incidence of aspiration pneumonia in community- and hospital-acquired pneumonia in hospitalized patients: a multicenter, prospective study in Japan. J Am Geriatr Soc.

