# Supplementary material for: Trends in the incidence and outcome of sepsis using data from a Japanese nationwide medical claims database-the Japan Sepsis Alliance (JaSA) study group-
Source: Crit Care. 2021 Sep 16;25:338. doi: 10.1186/s13054-021-03762-8 (PMC8444487; doi:10.1186/s13054-021-03762-8)
Supplement: Supplementary file 1 — Additional file 1: Table S1. Comorbidity categories with corresponding ICD-10. Table S2.. Focus of infection with corresponding ICD-10. Table S3. Acute organ dysfunction categories with corresponding ICD-10. Table S4. Demographics and clinical characteristics of patients with sepsis. Table S5. Focus of infection. Table S6. Organ support or dysfuntion. [file 13054_2021_3762_MOESM1_ESM.docx]

Trends in the incidence and outcome of sepsis using data from a Japanese nationwide medical claims database -the Japan Sepsis Alliance (JaSA) study group-

Taro Imaeda, Taka-aki Nakada, Nozomi Takahashi, Yasuo Yamao, Satoshi Nakagawa

Hiroshi Ogura, Nobuaki Shime, Yutaka Umemura, Asako Matsushima, Kiyohide Fushimi,

***Online data Supplement***

**Table S1.** Comorbidity categories with corresponding ICD-10

| Comorbidity | ICD-10 codes |
| --- | --- |
| Malignant tumor | C00-C97, D00-D09 |
| Hypertension | I10-I15 |
| Diabetes mellitus | E10-E14 |
| Heart failure | I50 |
| Cerebrovascular disease | I60-I69 |
| Ischemic heart disease | I20-I25 |
| Chronic respiratory disease | J40-J47 |
| Chronic renal failure | N18 |

**Table S2.** Focus of infection with corresponding ICD-10

| Focus of infection | ICD-10 codes |
| --- | --- |
| Respiratory | A15-A16, J00-J06, J09-J18, J20-J22, J31-J32, J35-J37, J39.0, J39.1, J85-J86 |
| Urogenital | A18.1, A51.0, A54.0-A54.2, A56.0-A56.2, A59.0, A60.0, N30.0, N30.8, N39.0, N41.0-N41.3, N45, N49.0-N49.2, N70-N77, O23 |
| Abdominal | A00-A09, A18.3, A42.1, A74.8, K35-K38, K57.0, K57.2, K57.4, K57.8, K61, K63.0, K63.1, K65, K67, K75.0, K80.0, K80.1, K80.3, K80.4, K81, K83.0 |
| Bone and soft tissue | A18.0, A18.4, A26.0, A28.1, A31.1, A31.8, A32.0, A36.3, A42.2, A43.1, A46, A48.0, L00-L08, M00, M01.0, M46.3, M46.5, M49.1-M49.3, M60.0, M86.0, M86.1, M86.65, M86.66, M86.69, M86.99 |
| Blood | A19 (Miliary tuberculosis), A40.0 (Invasive group A streptococcal disease), A49.0 (Methicillin susceptible Staphylococcus aureus bacteremia), A49.1 (Invasive pneumococcal disease), A49.9 (bacteremia) |

**Table S3.** Acute organ dysfunction categories with corresponding ICD-10

| Organ dysfunction | ICD-10 codes |
| --- | --- |
| Renal | N00.9（Acute nephritis syndrome） |
|  | N10（Acute tubulointerstitial nephritis） |
|  | N17.0（Shock kidney, Acute parenchymal renal failure, Acute kidney tubular necrosis） |
|  | N17.1（Acute renal cortical necrosis） |
|  | N17.8（Acute prerenal failure） |
|  | N17.9（Acute kidney injury） |
| Hepatic | K72.0（Acute liver failure） |
|  | K72.9（Liver failure, details unknown） |
|  | K76.8（Shock liver） |
| Thrombocytopenia | D69.5（Secondary thrombocytopenia） |
|  | D69.6（Thrombocytopenia） |
| Coagulopathy | D65（Disseminated intravascular coagulation） |
|  | D68.9（Other and unspecified coagulation disorders） |
| Acidosis | E87.2（Acidosis, metabolic or lactic） |

**Table S4.** Demographics and clinical characteristics of patients with sepsis

| Year | 2010 | 2011 | 2012 | 2013 | 2014 | 2015 | 2016 | 2017 | *P* value  for trend |
| --- | --- | --- | --- | --- | --- | --- | --- | --- | --- |
| Screened total inpatients, n | 3,820,159 | 4,873,980 | 6,274,524 | 6,257,095 | 7,130,432 | 7,261,192 | 7,555,957 | 7,316,789 | 0.0015 |
| Extracted sepsis, n | 110,469 | 162,809 | 206,909 | 231,488 | 297,432 | 323,682 | 354,451 | 355,833 | <0.0001 |
| Community-onset sepsis, n (%) | 66,635 (60.3) | 102,081 (62.7) | 130,054 (62.9) | 155,869 (67.3) | 212,571 (71.5) | 236,370 (73.0) | 263,609 (74.4) | 266,414 (74.9) | <0.0001 |
| Age, year ^a^ | 75 (65–83) | 75 (65–83) | 75 (64–83) | 75 (65–83) | 75 (66–84) | 76 (66–84) | 77 (67–84) | 77 (68–85) | 0.0005 |
| Female, n (%) | 44,488 (40) | 65,443 (40) | 82,625 (40) | 93,680 (40) | 124,058 (42) | 135,026 (42) | 149,176 (42) | 150,335 (42) | 0.0013 |
| Comorbidity |  |  |  |  |  |  |  |  |  |
| Malignant tumor, n (%) | 40,651 (36.8) | 58,997 (36.2) | 77,720 (37.6) | 85,029 (36.7) | 103,144 (34.7) | 110,819 (34.2) | 117,273 (33.1) | 118,586 (33.4) | 0.0038 |
| Hypertension, n (%) | 26,725 (24.2) | 40,556 (24.9) | 51,163 (24.7) | 58,858 (25.4) | 78,512 (26.4) | 84,708 (26.2) | 97,975 (27.7) | 99,321 (28.0) | 0.0038 |
| Diabetes mellitus, n (%) | 22,670 (20.5) | 35,221 (21.6) | 43,473 (21.0) | 49,590 (21.4) | 64,574 (21.7) | 71,499 (22.1) | 78,454 (22.2) | 79,957 (22.5) | 0.0016 |
| Heart failure, n (%) | 19,837 (18.0) | 30,481 (18.7) | 36,677 (17.7) | 40,861 (17.7) | 54,184 (18.2) | 60,160 (18.6) | 66,530 (18.8) | 67,946 (19.1) | 0.09 |
| Cerebrovascular disease, n (%) | 15,909 (14.4) | 23,945 (14.7) | 29,304 (14.2) | 32,663 (14.1) | 43,304 (14.6) | 46,504 (14.4) | 50,777 (14.3) | 50,231 (14.1) | 0.33 |
| Ischemic heart disease, n (%) | 11,683 (10.6) | 17,518 (10.8) | 21,853 (10.6) | 24,382 (10.5) | 31,434 (10.6) | 33,976 (10.5) | 37,449 (10.6) | 37,573 (10.6) | 0.36 |
| Chronic respiratory disease, n (%) | 9,293 (8.4) | 14,281 (8.8) | 17,344 (8.4) | 20,086 (8.7) | 26,853 (8.8) | 29,369 (9.0) | 31,832 (9.0) | 30,840 (8.7) | 0.09 |
| Chronic renal failure, n (%) | 4,800 (4.3) | 6,808 (4.2) | 8,721 (4.2) | 9,231 (4.0) | 12,070 (4.1) | 12,671 (3.9) | 12,854 (3.6) | 12,207 (3.4) | 0.09 |
| Focus of infection |  |  |  |  |  |  |  |  |  |
| Eligible patients, n | 67,318 | 100,060 | 126,414 | 141,670 | 181,813 | 197,388 | 227,876 | 233,449 | <0.0001 |
| Respiratory, n (%) | 26,563 (39.5) | 40,038 (40.0) | 48,027 (38.0) | 54,083 (38.2) | 72,420 (39.8) | 77,825 (39.4) | 99,085 (43.5) | 105,016 (45.0) | <0.0001 |
| Urogenital, n (%) | 9,911 (14.7) | 14,802 (14.8) | 19,223 (15.2) | 22,373 (15.8) | 29,328 (16.1) | 32,279 (16.4) | 34,034 (14.9) | 32,849 (14.1) | 0.99 |
| Abdominal, n (%) | 7,837 (11.6) | 11,453 (11.4) | 15,404 (12.2) | 17,920 (12.6) | 21,690 (11.9) | 23,777 (12.0) | 24,272 (10.7) | 25,056 (10.7) | 0.23 |
| Bone and soft tissue, n (%) | 3,607 (5.4) | 5,651 (5.6) | 7,099 (5.6) | 7,593 (5.4) | 10,264 (5.4) | 11,266 (5.7) | 12,542 (5.5) | 12,555 (5.4) | 0.23 |
| Blood, n (%) | 190 (0.3) | 284 (0.3) | 335 (0.3) | 445 (0.3) | 522 (0.3) | 553 (0.3) | 639 (0.3) | 592 (0.3) | 0.42 |
| Others, n (%) ^b^ | 2,368 (3.5) | 3,375 (3.4) | 4,399 (3.5) | 4,909 (3.5) | 6,264 (3.4) | 6,481 (3.3) | 6,954 (3.1) | 7,092 (3.0) | 0.0075 |
| Unknown, n (%) | 16,842 (25.0) | 24,457 (24.4) | 31,928 (25.3) | 34,347 (24.2) | 41,325 (22.7) | 45,207 (22.9) | 50,350 (22.1) | 50,289 (21.5) | 0.0007 |
| The hospital day of the blood  culture collection, day ^a^ | 1 (1–3) | 1 (1–3) | 1 (1–3) | 1 (1–2) | 1 (1–2) | 1 (1–2) | 1 (1–2) | 1 (1–2) | 0.0053 |
| Antibiotic treatment |  |  |  |  |  |  |  |  |  |
| Length, days ^a^ | 12 (8–21) | 12 (8–21) | 13 (8–22) | 12 (8–21) | 12 (8–21) | 12 (8–21) | 12 (8–21) | 12 (8–21) | 0.25 |
| Antibiotic-free days ^a^ | 16 (6–21) | 16 (7–21) | 16 (7–21) | 16 (7–21) | 17 (10–21) | 17 (10–21) | 16 (8–21) | 16 (8–20) | 0.27 |
| Length of hospital stay, days |  |  |  |  |  |  |  |  | 0.0002 |
| Mean (SD) | 51.3 (103.5) | 52.4 (104.0) | 51.5 (106.8) | 48.0 (114.1) | 45.0 (104.6) | 43.7 (100.0) | 42.3 (107.3) | 41.9 (99.9) |  |
| Median (IQR) | 34 (18–64) | 33 (17–63) | 33 (17–62) | 31 (16–57) | 29 (15–53) | 28 (15–52) | 27 (15–50) | 27 (15–50) |  |
| ICU admission, n (%) | 17,726 (16.0) | 26,535 (16.3) | 38,674 (18.7) | 42,062 (18.2) | 52,575 (17.7) | 55,631 (17.2) | 58,693 (16.6) | 58,469 (16.4) | 0.96 |
| Length of ICU stay, days |  |  |  |  |  |  |  |  | 0.023 |
| Mean (SD) | 6.9 (5.9) | 7.1 (6.3) | 7.2 (6.4) | 7.1 (6.5) | 6.8 (6.1) | 6.8 (6.0) | 6.7 (5.9) | 6.7 (6.0) |  |
| Median (IQR) | 5 (2–12) | 5 (2–12) | 6 (2–12) | 5 (2–12) | 5 (2–11) | 5 (2–11) | 5 (2–11) | 5 (2–11) |  |
| In-hospital mortality, n (%) ^c^ | 25,120 (24.8) | 33,668 (23.0) | 41,649 (22.5) | 43,021 (20.9) | 51,425 (19.6) | 53,826 (18.9) | 57,749 (18.5) | 56,905 (18.3) | <0.0001 |

^a^ The data shown represent the median value along with the interquartile range.

^b^ Others include meninges, brain, spinal cord, heart, and sexually transmitted disease.

^c^ After excluding repeat hospitalizations, annual numbers of patients with sepsis were 101,307 in 2010, 146,096 in 2011, 185,227 in 2012, 206,294 in 2013, 262,332 in 2014, 284,247 in 2015, 311,195 in 2016, and 311,415 in 2017.

ICU: intensive care unit; SD: standard deviation; IQR: interquartile range

| Year | 2010 | 2011 | 2012 | 2013 | 2014 | 2015 | 2016 | 2017 | *P* value  for trend |
| --- | --- | --- | --- | --- | --- | --- | --- | --- | --- |
| N.* | 67,318 | 100,060 | 126,414 | 141,670 | 181,813 | 197,388 | 227,876 | 233,449 | <0.0001 |
| Lung/lower respiratory tract, n (%) | 24,363 (36.2) | 36,835 (36.8) | 43,759 (34.6) | 49,460 (34.9) | 66,635 (36.7) | 71,810 (36.4) | 92,302 (40.5) | 98,318 (42.1) | 0.036 |
| Intestine, n (%) | 6,207 (9.2) | 9,276 (9.3) | 12,257 (9.7) | 13,475 (9.5) | 16,292 (9.0) | 17,406 (8.8) | 18,847 (8.3) | 18,282 (7.8) | 0.011 |
| Biliary tract, n (%) | 4,081 (6.1) | 6,073 (6.1) | 8,015 (6.3) | 9,469 (6.7) | 11,545 (6.3) | 12,780 (6.5) | 13,145 (5.8) | 13,829 (5.9) | 0.52 |
| Kidney/urinary tract, n (%) | 3,303 (4.9) | 4,967 (5.0) | 6,169 (4.9) | 7,970 (5.6) | 11,861 (6.5) | 13,599 (6.8) | 13,998 (6.1) | 13,292 (5.7) | 0.052 |
| Skin/soft tissue, n (%) | 3,237 (4.8) | 5,047 (5.0) | 6,272 (5.0) | 6,652 (4.7) | 8,990 (4.9) | 9,933 (5.0) | 11,057 (4.9) | 11,075 (4.7) | 0.58 |
| Intraperitoneal, n (%) | 2,694 (4.0) | 3,856 (3.9) | 5,338 (4.2) | 5,962 (4.2) | 7,271 (4.0) | 7,872 (4.0) | 7,905 (3.5) | 7,961 (3.4) | 0.060 |
| Oral cavity/pharynx/nasal  cavity/neck, n (%) | 1,678 (2.5) | 2,452 (2.5) | 3,317 (2.6) | 3,611 (2.5) | 4,466 (2.5) | 4,579 (2.3) | 5,385 (2.4) | 5,407 (2.3) | 0.027 |
| Meninges/brain/spinal cord, n (%) | 1,419 (2.1) | 2,086 (2.1) | 2,719 (2.2) | 3,081 (2.2) | 3,876 (2.1) | 4,112 (2.1) | 4,343 (1.9) | 4,348 (1.9) | 0.052 |
| Liver, n (%) | 1,062 (1.6) | 1,524 (1.5) | 2,051 (1.6) | 2,489 (1.8) | 2,874 (1.6) | 3,125 (1.6) | 3,222 (1.4) | 3,266 (1.4) | 0.24 |
| Heart, n (%) | 732 (1.1) | 993 (1.0) | 1,305 (1.0) | 1,416 (1.0) | 1,871 (1.0) | 1,818 (0.9) | 1,941 (0.9) | 2,063 (0.9) | 0.0019 |
| Pleural cavity, n (%) | 522 (0.8) | 751 (0.8) | 951 (0.8) | 1,012 (0.7) | 1,319 (0.7) | 1,436 (0.7) | 1,398 (0.6) | 1,291 (0.6) | 0.0019 |
| Bone and joints, n (%) | 370 (0.5) | 604 (0.6) | 827 (0.7) | 941 (0.7) | 1,274 (0.7) | 1,333 (0.7) | 1,485 (0.7) | 1,480 (0.6) | 0.25 |
| Reproductive organs, n (%) | 401 (0.6) | 559 (0.6) | 797 (0.6) | 928 (0.7) | 1,175 (0.6) | 1,274 (0.6) | 1,189 (0.5) | 1,275 (0.5) | 0.25 |
| Blood, n (%) | 190 (0.3) | 284 (0.3) | 335 (0.3) | 445 (0.3) | 522 (0.3) | 553 (0.3) | 639 (0.3) | 592 (0.3) | 0.42 |
| Sexually transmitted disease, n (%) | 217 (0.3) | 296 (0.3) | 375 (0.3) | 412 (0.3) | 517 (0.3) | 551 (0.3) | 670 (0.3) | 681 (0.3) | 0.08 |
| Unknown, n (%) | 16,842 (25.0) | 24,457 (24.0) | 31,928 (25.0) | 34,347 (24.0) | 41,325 (22.7) | 45,207 (22.9) | 50,350 (22.1) | 50,289 (21.5) | 0.0007 |

**Table S5.** Focus of infection

* The number of cases in which the name of the infectious disease was registered in the Diagnosis Procedure Combination data

**Table S6.** Organ support or dysfunction

| Year | 2010 | 2011 | 2012 | 2013 | 2014 | 2015 | 2016 | 2017 | *P* value  for trend |
| --- | --- | --- | --- | --- | --- | --- | --- | --- | --- |
| Extracted sepsis, n | 110,469 | 162,809 | 206,909 | 231,488 | 297,432 | 323,682 | 354,451 | 355,833 | <0.0001 |
| Vasopressor use, n (%) | 12,609 (11.4) | 20,083 (12.3) | 29,281 (14.2) | 29,879 (12.9) | 35,794 (12.0) | 38,558 (11.9) | 43,660 (12.3) | 43,164 (12.1) | 0.81 |
| Ventilator use, n (%) | 20,171 (18.3) | 29,754 (18.3) | 40,418 (19.5) | 42,793 (18.5) | 52,629 (17.7) | 56,013 (17.3) | 60,204 (17.0) | 60,139 (16.9) | 0.021 |
| Length of mechanical  ventilation, days * | 8 (3-18) | 8 (3-18) | 7 (3-18) | 7 (3-17) | 7 (3-16) | 7 (3-15) | 6 (3-15) | 6 (2-14) | 0.041 |
| Oxygen therapy, n (%) | 73,204 (66.3) | 107,482 (66.0) | 132,922 (64.2) | 149,542 (64.6) | 192,969 (64.9) | 210,863 (65.1) | 232,591 (65.6) | 232,887 (65.4) | 0.64 |
| Length of oxygen therapy,  days * | 7 (3-16) | 7 (3-16) | 7 (3-16) | 6 (3-15) | 6 (2-14) | 6 (2-14) | 6 (2-13) | 6 (2-13) | 0.0001 |
| RRT use, n (%) | 7,812 (7.1) | 11,590 (7.1) | 15,572 (7.5) | 16,235 (7.0) | 19,691 (6.6) | 20,938 (6.5) | 22,143 (6.2) | 22,382 (6.3) | 0.0055 |
| Length of RRT, days * | 7 (3-14) | 7 (3-14) | 7 (3-14) | 7 (3-14) | 7 (3-14) | 7 (3-14) | 7 (3-13) | 7 (3-13) | 0.053 |
| Acute organ dysfunction categories with corresponding ICD-10 | | | | | | | | |  |
| Renal dysfunction, n (%) | 54,697 (49.5) | 79,459 (48.8) | 100,145 (48.4) | 107,835 (46.6) | 133,591 (44.9) | 140,982 (43.6) | 148,017 (41.8) | 148,264 (41.7) | <0.0001 |
| Hepatic dysfunction, n (%) | 4,473 (4.0) | 6,392 (3.9) | 8,212 (4.0) | 9,034 (3.9) | 11,149 (3.7) | 12,287 (3.8) | 13,134 (3.7) | 12,693 (3.6) | 0.0022 |
| Thrombocytopenia / Coagulopathy, n (%) | 14,927 (13.5) | 21,545 (13.2) | 28,628 (13.8) | 30,606 (13.2) | 33,132 (11.1) | 34,335 (10.6) | 36,722 (10.4) | 35,515 (10.0) | 0.0012 |
| Acidosis, n (%) | 697 (0.6) | 1,067 (0.7) | 1,329 (0.6) | 1,587 (0.7) | 2,161 (0.7) | 2,403 (0.7) | 3,030 (0.9) | 3,318 (0.9) | 0.0074 |

* The data shown represent the median value along with the interquartile range.

RRT: renal replacement therapy
