# Supplementary material for: Trends in the incidence and outcome of sepsis using data from a Japanese nationwide medical claims database-the Japan Sepsis Alliance (JaSA) study group-
Source: Crit Care. 2021 Sep 16;25:338. doi: 10.1186/s13054-021-03762-8 (PMC8444487; doi:10.1186/s13054-021-03762-8)
Supplement: Supplementary file 2 — Additional file 1: Figure S1. Annual change in the number of patients with sepsis and deaths in sepsis. [file 13054_2021_3762_MOESM2_ESM.pdf]

Figure S1. Annual change in the number of patients with sepsis and deaths in sepsis

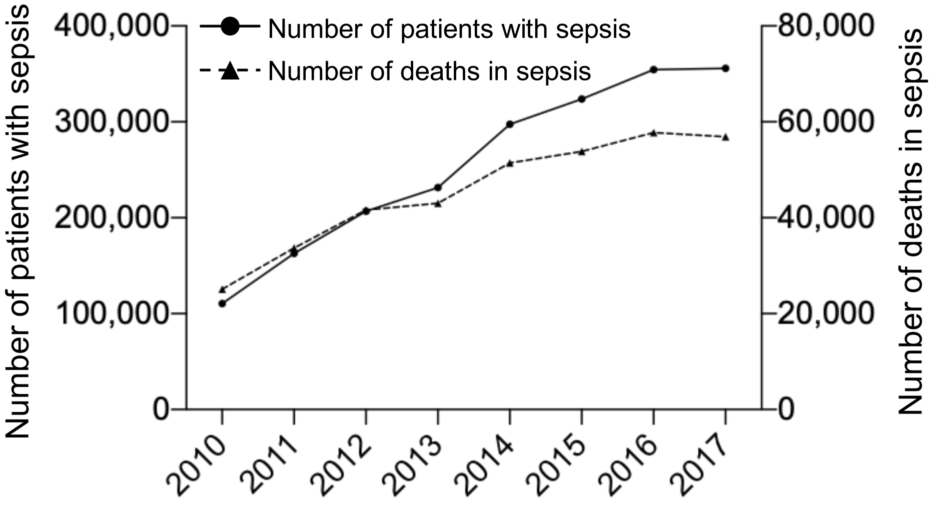

Number of patients with sepsis: +36,810/y [95% CI, +30,050 to +43,564], R^2=0.97, P<0.0001

Number of deaths in sepsis: +5,138/y [95% CI, +3,781 to +6,495], R^2=0.93, P<0.0001

Error bars indicate 95% CI. CI, confidence interval
