# Supplementary material for: Trends in the incidence and outcome of sepsis using data from a Japanese nationwide medical claims database-the Japan Sepsis Alliance (JaSA) study group-
Source: Crit Care. 2021 Sep 16;25:338. doi: 10.1186/s13054-021-03762-8 (PMC8444487; doi:10.1186/s13054-021-03762-8)
Supplement: Supplementary file 3 — Additional file 1: Figure S2a. Annual change in the in-hospital mortality rate according to the age subgroups by dividing into units of 10 years. [file 13054_2021_3762_MOESM3_ESM.pdf]

Figure S2a. Annual change in the in-hospital mortality rate according to the age subgroups by dividing into units of 10 years

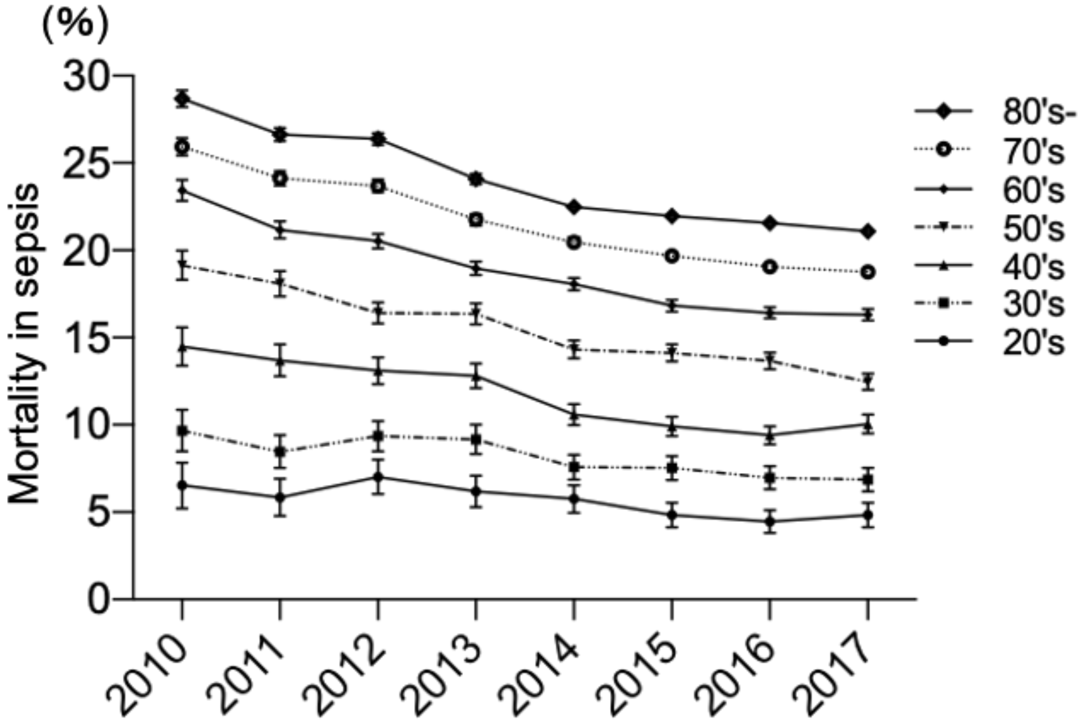

≥80 years (80's-): -1.11%/y [95% CI, -1.40% to -0.83%], R^2=0.94, P<0.0001
70–79 years (70's): -1.06%/y [95% CI, -1.27% to -0.84%], R^2=0.96, P<0.0001
60–69 years (60's): -1.02%/y [95%CI, -1.27% to -0.77%], R^2=0.94, P<0.0001
50–59 years (50's): -0.93%/y [95%CI, -1.11% to -0.74%], R^2=0.96, P<0.0001
40–49 years (40's): -0.77%/y [95% CI, -1.02% to -0.51%], R^2=0.90, P=0.0003
30–39 years (30's): -0.41%/y [95%CI, -0.61% to -0.21%], R^2=0.81, P=0.0024
20–29 years (20's): -0.31%/y [95%CI, -1.51% to -0.10%], R^2=0.69, P=0.011
Error bars indicate 95% CI. CI: confidence interval
