# Supplementary material for: Trends in the incidence and outcome of sepsis using data from a Japanese nationwide medical claims database-the Japan Sepsis Alliance (JaSA) study group-
Source: Crit Care. 2021 Sep 16;25:338. doi: 10.1186/s13054-021-03762-8 (PMC8444487; doi:10.1186/s13054-021-03762-8)
Supplement: Supplementary file 4 — Additional file 1: Figure S2b. Annual change in the length of hospital stay according to the age subgroups by dividing into units of 10 years. [file 13054_2021_3762_MOESM4_ESM.pdf]

Figure S2b. Annual change in the length of hospital stay according to the age subgroups by dividing into units of 10 years

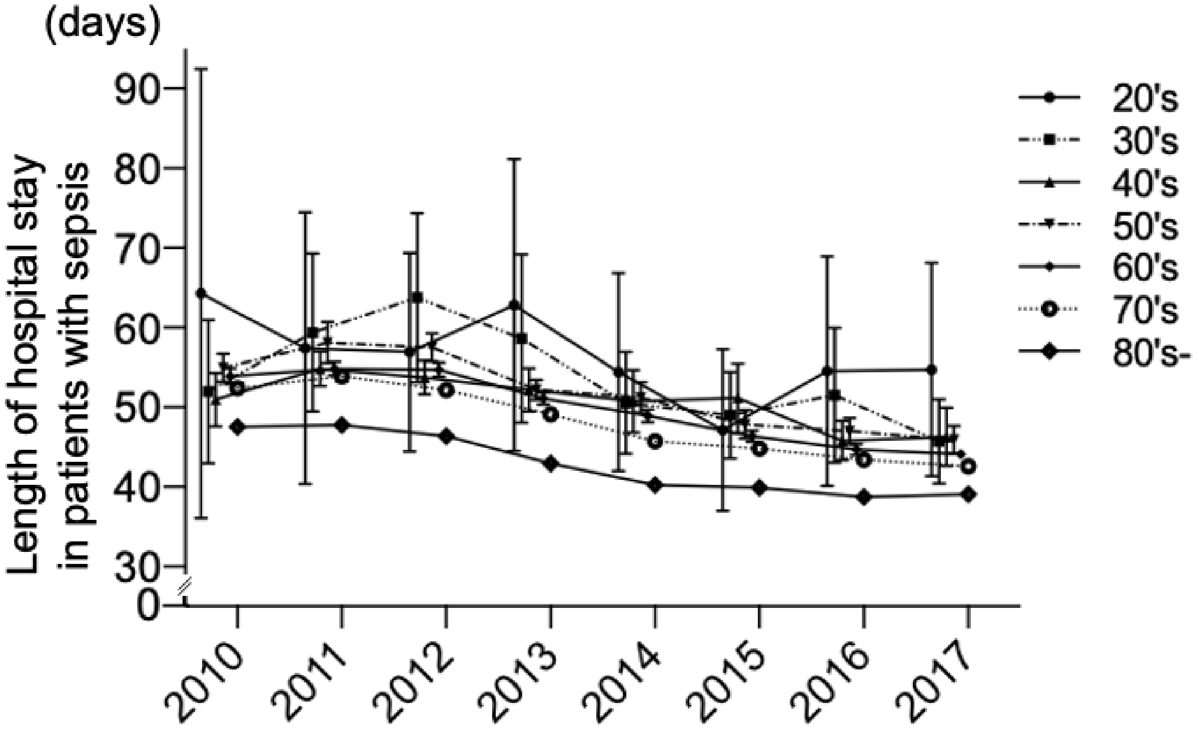

20–29 years (20's): -1.42%/y [95% CI, -3.08% to 0.25%], R^2=0.42, *P*=0.082
30–39 years (30's): -1.62%/y [95%CI, -3.52% to 0.29%], R^2=0.42, *P*=0.083
40–49 years (40's): -1.04%/y [95%CI, -1.84% to -0.23%], R^2=0.62, *P*=0.020
50–59 years (50's): -1.77%/y [95% CI, -2.53% to -1.01%], R^2=0.84, *P*=0.0013
60–69 years (60's): -1.75%/y [95%CI, -2.27% to -1.23%], R^2=0.92, *P*=0.0002
70–79 years (70's): -1.75%/y [95%CI, -2.25% to -1.25%], R^2=0.92, *P*=0.0001
≥80 years (80's-): -1.50%/y [95%CI, -1.99% to -1.02%], R^2=0.90, *P*=0.0003
Error bars indicate 95% CI. CI: confidence interval
